# Tryptophan depletion in context of the inflammatory and general nutritional status of a low-income South African HIV-infected population

**DOI:** 10.1186/s41043-016-0042-4

**Published:** 2016-02-17

**Authors:** Priyesh Bipath, Peter F. Levay, Margaretha Viljoen

**Affiliations:** 1 Department of Physiology, School of Medicine, Faculty of Health Sciences, University of Pretoria, Pretoria, South Africa; 2 Department of Internal Medicine (Kalafong Hospital), School of Medicine, Faculty of Health Sciences, University of Pretoria, Pretoria, South Africa; 3 Department of Psychiatry, School of Medicine, Faculty of Health Sciences, University of Pretoria, Private Bag X323, 0007 Pretoria, South Africa

**Keywords:** Tryptophan, HIV/AIDS, Malnutrition, Sub-Saharan, Pro-inflammatory activity

## Abstract

**Background:**

The essential amino acid tryptophan cannot be synthesised in the body and must be acquired through dietary intake. Oxidation of tryptophan, due to immune induction of the enzyme indoleamine 2,3-dioxygenase (IDO), is considered to be the main cause of tryptophan depletion in HIV infection and AIDS.

We examined plasma tryptophan levels in a low-income sub-Saharan HIV-infected population and compared it to that of developed countries. Tryptophan levels were further examined in context of the general nutritional and inflammatory status.

**Methods:**

This cross-sectional study included 105 HIV-positive patients recruited from the Kalafong Hospital in Pretoria, South Africa, and 60 HIV-negative controls.

**Results:**

Patient tryptophan levels were in general markedly lower than those reported for developed countries. In contrast to reports from developed countries that showed tryptophan levels on average to be 18.8 % lower than their control values, tryptophan levels in our study were 44.1 % lower than our controls (24.4 ± 4.1 vs. 43.6 ± 11.9 μmol/l; *p* < 0.001). Tryptophan levels correlated with both CD4 counts (*r* = 0.341; *p* = 0.004) and with pro-inflammatory activity as indicated by neopterin levels (*r* = −0.399; *p* = 0.0001). Nutritional indicators such as albumin and haemoglobin correlated positively with tryptophan and negatively with the pro-inflammatory indicators neopterin, interleukin 6 and C-reactive protein. The most probable causes of the lower tryptophan levels seen in our population are food insecurity and higher levels of inflammatory activity.

**Conclusions:**

We contend that inflammation-induced tryptophan depletion forms part of a much wider effect of pro-inflammatory activity on the nutritional profile of HIV-infected patients.

## Background

Tryptophan, as an essential amino acid, cannot be synthesised in the body and must be acquired through intake and from tryptophan released during protein turnover [1]. A daily nutritional intake at around 20 mmol is said to be required to sustain normal plasma levels [2]. The majority of tryptophan is utilised for the synthesis of tissue proteins [3], whereas about 1 % of dietary tryptophan serves as a substrate for the biosynthesis of serotonin [2, 4]. Tryptophan is also used for the de novo synthesis of niacin and is said to play a role in the immune regulation of normal T-cell function [5–7]. Excess tryptophan, i.e., at levels above the requirement for protein and serotonin synthesis, is oxidised in the liver under influence of the liver-specific enzyme l-tryptophan 2,3-dioxygenase (TDO), to ATP, CO_2_, and water. In contrast, tryptophan oxidation under influence of the inflammation-inducible enzyme indoleamine 2,3-dioxygenase (IDO) occurs in various cell types and is not limited by a decrease in tryptophan levels [6, 7]. Two forms of indoleamine 2,3-dioxygenase exist, i.e., indoleamine 2,3-dioxygenase-1 and indoleamine 2,3-dioxygenase-2, with subtle differences in substrate specificity and inhibition characteristics [7].

Tryptophan depletion in HIV infection and AIDS has been described previously [6, 8–20]. However, these results are generally based on populations from developed countries. In developed countries, oxidation of tryptophan along the kynurenine pathway, under influence of the rate-limiting enzyme IDO, is considered to be the main cause of tryptophan depletion in HIV and AIDS [5, 6, 8, 9]. This study examined tryptophan levels in a black, low-income population from the Gauteng Province, South Africa, and compared it to that reported for populations from developed countries. The study further examined tryptophan depletion in context of the inflammatory and general nutritional status of HIV-infected patients.

## Methods

A group of 105 HIV-positive adult (>18 years old) black patients were recruited from the Immunology Clinic at Kalafong Secondary Hospital which provides health services predominantly to communities of Atteridgeville and surrounding areas west of Pretoria. Although the patients lived predominantly in Atteridgeville, they were mostly born outside of the township and it is estimated that about one third are immigrants from other African countries. They are generally of low income, with poor housing, and many are unemployed. Some families survive on a single grant or pension. Maize meal is the staple diet of the population, but a tendency to convert to fast foods occurs when their socio-economic status allows. Sixty-four percent (64 %) of the highly active antiretroviral treatment (HAART) group, 60 % of the HAART naïve group and 63 % of the control group were female. The mean ages of the different groups (HAART 37.9 ± 8.9; naïve 37.1 ± 10.2; and controls 31.2 ± 8.1 years) were not statistically different.

Patients were on antiretroviral treatment for an average period of 15.9 ± 16.5 months. At the time of sample collection, triple therapy was given, mostly efavirenz (EFV)/nevaripine (NVP), lamuvidine (3TC) and stavudine (D4T). Presently, the clinic is converting to the FDC (fixed drug combination: emtricitabine, tenofovir, lamuvidine), single dose with apparently better patient compliance. At baseline, 22.9 % of the total HIV patient group were confirmed by sputum smears as TB positive. TB-positive patients were treated with isoniazid (INH), pyrazinamide (PZA), rifampicin (RIF) and ethambutol (ETH) for at least 6 months depending on the sensitivity of the TB organism. Although patients are initially educated as to a well-balanced healthy diet and their weights are monitored, they do not have regular one-on-one counselling with a dietician. The HIV patients were divided into HAART (*n* = 75) and HAART naïve (*n* = 30) groups. Patients were also subdivided according to co-infection with TB (*n* = 24). Sixty HIV-negative controls were recruited from the South African National Blood Service (SANBS) satellite site based in Pretoria West.

This was a cross-sectional study based on the analysis of patients’ blood samples. The study received ethical approval, in accordance with the Declaration of Helsinki, from the Faculty of Health Sciences Research and Ethics Committee of the University of Pretoria (107/2008) and from the South African National Blood Service Human Research Committee (2010/03). Written or verbal informed consent, as witnessed and formally recorded, was obtained from all volunteers prior to participation in this study. The study was conducted over a period of 2 years from January 2012.

Although patients were not instructed to fast overnight, blood was drawn in the morning during the patients’ scheduled visit to the clinic. Plasma samples for neopterin, IL-6, interferon-γ and tryptophan were processed on site and stored at −70 °C until analysis. Tryptophan was quantified by gas chromatography mass spectrometry (GC-MS) using a method developed and validated in our laboratory. Briefly, samples were processed and derivatized with pentafluoropropionic anhydride and pentafluoropropanol before analysis. Isotope label deuterated tryptophan was used as the internal standard. The GC oven was programmed to begin at an initial temperature of 80 °C with a ramp at a rate of 20 °C up to 180 °C followed by a 10 °C ramp up to a maximum temperature of 280 °C. Sample peaks were eluted on a DB-5MS capillary column within a chromatographic runtime of 18 min using a Thermo Scientific Trace 1300 gas chromatographer coupled to an ISQ single quadropole mass spectrometer.

Neopterin was measured as the primary indicator of inflammatory activity. Neopterin, a catabolic product of the purine nucleotide guanosine triphosphate, is produced from guanosine 5′-triphosphate (GTP) that is cleaved by GTP cyclohydrolase 1 to 7,8-dihydroneopterin triphosphate, followed by conversion of 7,8-dihydroneopterin triphosphate to neopterin and 7,8-dihydroneopterin under the influence of phosphatases [21]. GTP cyclohydrolase 1 is stimulated, predominantly, by T-helper cell type-1-derived interferon-γ, but co-stimulation by tumour necrosis factor alpha may contribute [21]. Neopterin has been shown, both in our laboratories and in that of others, to be an excellent indicator of pro-inflammatory activity [13, 21, 22].

Neopterin was measured by sandwich ELISA according to the manufacturer’s kit protocol (Immuno-Biological Laboratories Inc., USA). The pro-inflammatory cytokines IL-6 and interferon-γ (IFN-γ) were determined using a cytometric bead array kit (BD Biosciences, San Jose, CA, USA) and flow cytometry. All other laboratory variables were determined by the National Health Laboratory service (NHLS) at Kalafong.

Group comparisons were assessed by analysis of variance (ANOVA) following data collation testing for normality and log transformations. Because the data did not show a normal distribution, the non-parametric Kruskal-Wallis test was employed for comparisons between subgroups. Actual values are expressed as mean and standard deviation. Non-parametric Spearman rank correlation coefficients were used to determine associations between group variables. All analyses were performed at a significance level of *p* < 0.05 using Statistical Package for Social Sciences version 22 (SPSS, IBM, Endicott, NY, USA).

## Results

The total patient group presented with a mean viral load of 2.8 ± 1.4 log_10_ copies/ml and a CD4 count of 258.0 ± 193.1 cells/μl. The tryptophan level for the total patient group (24.4 ± 4.1 μmol/l) was significantly (*p* < 0.0001) lower than that of the control group (43.6 ± 11.9 μmol/l). Patient tryptophan levels were thus almost half (44.1 %) of that of the controls. Patients with a CD4 count of less than 200 cells/μl presented with significantly lower tryptophan levels (23.0 ± 4.2 vs. 26.1 ± 4.1 μmol/l; *p* = 0.03) than patients with CD4 counts greater than 200 cells/μl. The tryptophan levels in the HAART naive group (22.0 ± 4.3 μmol/l) were significantly (*p* = 0.03) lower than that of the HAART group (25.1 ± 3.8 μmol/l). Tryptophan levels correlated with both CD4 count and neopterin values for the total patient (CD4 *r* = 0.341; *p* = 0.004, neopterin *r* = −0.399; *p* = 0.0001) and HAART (CD4 *r* = 0.291; *p* = 0.04, neopterin *r* = −0.359; *p* = 0.002) groups. Tryptophan levels were not significantly different between the HIV patients with TB (23.9 ± 4.9 μmol/l) and those without TB co-infection (24.5 ± 3.9 μmol/l).

Tryptophan levels were subsequently compared to that found in studies from developed countries (Table 1). Although values from developed countries varied, tryptophan levels in patients of our study were in general markedly lower.

**Table 1:** Comparison of tryptophan levels in HIV/AIDS patients of this study and that of developed countries

	A	B	C	D	*p* value
	Controls	Total group	ART—naïve	ART	
Present study (gc)	43.6 (*n* = 60)	24.4 (*n* = 105)	22.0 (*n* = 30)	25.1 (*n* = 75) (71.0% on ART)	<0.001 (A–B) <0.001 (A–C) <0.001 (A–D) 0.03 (C–D)
Developed countries:					
Werner et al. 1988 [10] (h)	91.0	44.8 (*n* = 11)	-	-	<0.0001 (A–C)
Larsson et al. 1989 [11] (h)	39.7 (*n* = 14)	28.4 (*n* = 24)	-	-	<0.05 (A–B)
Fuchs et al. 1990 [12] (h)	91.1 (*n* = 11)	48.8 dementia 70.5	-	-	<0.05 (A–B) <0.01 (A–B)
Fuchs et al. 1990 [9] (h)	39.7	29.8 (*n* = 22)	-	-	-
Fuchs et al. 1991 [13] (h)	91.0	57.0 (*n* = 42)	(62 % naïve)	(38 % on ART)	<0.01 (A–B)
Heyes et al. 1992 [14] (h)	70.9 (*n* = 20)	40.2 (*n* = 107)	-	-	<0.0001 (A–B)
Gisselen et al. 1994 [15] (h)	-	29.4 (*n* = 14)	29.4 (pre-ART)	36.2 (post-ART)	<0.01 (C–D)
Hortin et al. 1994 [16] (fl)	46 (*n* = 20)	22 (*n* = 20)	-	(85 % on ART)	<0.001 (A–B)
Laurichesse et al. 1998 [17] (fl)	59 (*n* = 8)	51 (*n* = 7)	-	-	<0.05 (A–B)
Huengsberg et al. 1998 [18] (h)	56.3 (*n* = 72)	50.1 (*n* = 82)	-	-	<0.01 (A–B)
Look et al. 2000 [19] (gc)	52.6 (*n* = 55)	44.6 (*n* = 17) 37.4 (*n* = 7) AIDS	44.6 (pre-ART)	53.0 (post-ART)	0.14 (A–B) 0.009 (A–B) 0.039 (C–D)
Murray et al. 2001 [20] (h)	49.3	69.2 (post niacin treatment)	-	-	-
Zangerle et al. 2002 [8] (h)	65.8 (*n* = 40)	44.1 (*n* = 45)	44.1 (pre-ART)	53.2 (post-ART)	<0.001 (A–B) <0.001 (A–D) 0.001 (C–D)
Schroeksnadel et al. 2008 [6] (h)	-	51.4 (*n* = 152)	-	(70 % on ART)	-

Analytical methods used: *gc* gas chromatography mass spectrometry, *fl* fluorometric procedure, *h* high-performance liquid chromatography

Albumin (29.0 ± 8.0 vs. 37.5 ± 3.9 g/l; *p* < 0.001), haemoglobin (10.7 ± 2.4 vs. 12.7 ± 1.9 g/dl; *p* = 0.001), BMI (21.5 ± 5.6 vs. 25.4 ± 7.1 kg/m^2^; *p* = 0.02) and albumin/globulin (A/G) ratio (0.5 ± 0.2 vs. 0.7 ± 0.2; *p* < 0.0001) were significantly lower in patients with CD4 counts less than 200 cells/μl. In Table 2, correlations for the total group of patients are shown between tryptophan, CD4 counts, neopterin, IL-6, and CRP levels, on the one hand, and albumin, globulin, the albumin/globulin (A/G) ratio, haemoglobin, red cell distribution width and body mass index (BMI), on the other.

**Table 2:** Correlations for the total group of patients between albumin, globulin, the albumin/globulin (A/G) ratio, haemoglobin, red cell distribution width and BMI, on the one hand, and tryptophan, CD4 counts, neopterin, IL-6, and CRP levels, on the other

	Tryptophan (μM)		CD4 count (cells/μl)		Neopterin (nmol/l)		IL-6 (pg/ml)		CRP (mg/l)	
	Rho	*p* value	Rho	*p* value	Rho	*p* value	Rho	*p* value	Rho	*p* value
Albumin (g/l)	0.357*	0.001	0.491*	<0.0001	−0.584*	<0.0001	−0.274*	0.009	−0.442*	<0.0001
Globulin (g/l)	−0.321*	0.001	−0.271*	0.020	0.351*	0.0004	0.177	0.074	0.378*	0.0002
A/G ratio	0.385*	0.0003	0.486*	<0.0001	−0.539*	<0.0001	−0.225*	0.033	−0.468*	<0.0001
Hb (g/dl)	0.378*	0.0003	0.420*	0.0003	−0.576*	<0.0001	−0.193	0.063	−0.250*	0.020
RDW (%)	−0.319*	0.003	−0.307*	0.001	0.334*	0.001	0.044	0.676	0.136	0.212
BMI (kg/m^2^)	0.154	0.157	0.368*	0.004	−0.317*	0.003	−0.360*	0.001	−0.066	0.573

*A/G* albumin to globulin ratio, *Hb* haemoglobin, *RDW* red cell distribution width, *BMI* body mass index

Spearman Rho correlation significant, *p < 0.05

Patients with CD4 less than 200 cells/μl presented with significantly elevated neopterin (70.5 ± 45.1 nmol/l vs. 24.1 ± 20.9; *p* < 0.0001) as compared to patients with CD4 counts greater than 200 cells/μl. The neopterin levels of HIV patients in the present study were subsequently compared to that reported in developed countries (Table 3).

**Table 3:** Neopterin levels in HIV patients compared to that of HIV patients from populations of developed countries

	Total patient groups		At lower CD4		At higher CD4	
Study	NPT	CD4	NPT	CD4	NPT	CD4
Present study	45.6	258.0 cells/μl	70.5	<200 cells/μl	24.1	>200 cells/μl
Schroeksnadel et al. 2008 [6]	14.1	404 cells/mm^3^	-	-	-	-
Zangerle et al. 2002 [8]	23.4	112 cells/μl	23.4	112 cells/μl	8.0	232 cells/μl
Mildvan et al. 2005 [57]	16.0	75 cells/ml	20.4	50 cells/ml	9.9	200 cells/ml
Hanna et al. 2009 [58]	-	-	24.4	<200 cells/μl	12.5	>200 cells/μl
Kurz et al. 2009 [59]	25.0	204 cells/mm^3^	-	-	-	-
Bogner et al. 1988 [60]	-	-	29.7	264 cells/μl	14.4	487 cells/μl

*NPT* neopterin (nmol/l)

## Discussion

Increased oxidation of tryptophan in the kynurenine pathway is considered the major cause of tryptophan depletion in HIV-infected and AIDS patients. Oxidative metabolism of tryptophan along the kynurenine pathway increases during inflammatory conditions and is regulated by the rate-limiting enzyme IDO which is stimulated by pro-inflammatory cytokines, predominantly IFN-γ [7, 22]. Evidence for decreased tryptophan levels in HIV patients from populations in developed countries has been shown by several studies (Table 1). However, a lack exists in information on tryptophan levels in HIV-infected AIDS populations from sub-Saharan regions.

The present study investigated tryptophan levels in context of the inflammatory and nutritional status in a low-income, black South African HIV-infected population. The results of this study confirmed tryptophan depletion in patients with HIV. However, tryptophan levels were markedly lower than that found in developed countries (Table 1). In contrast to reports from developed countries that showed tryptophan levels on average to be 18.8 % lower than control values (calculated from values in Table 1), patient tryptophan levels in the present study were 44.1 % lower. The degree of tryptophan depletion correlated with the decline in the CD4 count (*r* = 0.341; *p* = 0.005) and therefore with the degree of immune deficiency. No differences were seen between TB-positive and TB-negative HIV patients. However, TB-positive patients were all on anti-TB treatment.

The study showed, in line with studies from developed countries (Table 1), tryptophan to be significantly lower in the treatment naïve patients than in patients on HAART. In view of the role of inflammatory activity in the degradation of tryptophan and the lower levels of inflammation seen in the HAART than the HAART-naïve group, depicted by neopterin and perhaps IL-6 and CRP, it is reasonable to assume the higher tryptophan levels in HAART patients to be the results of a partial correction of the HIV/AIDS-induced inflammatory activity in patients treated with antiretrovirals. Tryptophan levels were subsequently compared to the degree of pro-inflammatory activity as reflected by levels of the pro-inflammatory cytokines IL-6, IFN-γ, the acute phase protein CRP and with neopterin. Tryptophan correlated inversely with neopterin (*r* = −0.399; *p* = 0.0001) and IL-6 (*r* = −0.230; *p* = 0.026). The decline in tryptophan levels with increases in inflammatory activity is in agreement with evidence from previous studies [8, 13]. Perhaps of greater importance is the fact that the decline in tryptophan levels correlated with increases in the levels of the pro-inflammatory cytokine IFN-γ (*r* = −0.217; *p* = 0.036), the major stimulus for indoleamine 2,3-dioxygenase.

Several factors may contribute to lower tryptophan levels in low-income sub-Saharan HIV-infected and AIDS populations when compared to levels found in populations from developed countries, the most important probably food insecurity, but a higher degree of inflammation could potentially also contribute.

### Food insecurity as a possible reason for tryptophan depletion

Malnutrition is considered a worldwide phenomenon in HIV, especially where it has progressed to AIDS [23]. Several factors in HIV infection and AIDS can potentially contribute to this state of malnutrition, including nutrient availability, gastrointestinal problems (such as diarrhoea, dysphagia, odynophagia, nausea, vomiting, gastrointestinal bleeding and neoplasias), suppressed appetite and altered metabolic processes such as increases in metabolic rate, increased protein catabolism and increases in nutritional requirements [23].

In addition to the conditions mentioned above for HIV-infected and AIDS patients in general, additional factors exist in low-income populations of developing countries that may influence the nutritional status and by implication, tryptophan levels in such patients. Malnutrition, irrespective of HIV status, is a common feature in many populations of Africa [24]. According to the 2012 Global Hunger Index, South Africa is ranked ninth in the world for highest hunger levels [25]. Major causes of the widespread malnutrition include limited food or financial resources and poor nutritional value of available food [24]. An additional contributing factor may be dietary changes upon urbanisation. South Africa is in a rural-to-urban transition phase, a phenomenon often associated with a shift towards energy-dense foods poor in proteins and amino acids [26, 27].

In developed countries, the dietary availability of tryptophan would appear not to be a major problem [5]. While the average daily intake of tryptophan in developed countries is estimated to be around 1 g, the estimated daily requirement is said to be between 175 and 250 mg [5]. This would leave a fair safety margin in HIV-infected and AIDS populations with adequate nutritional resources. However, the same cannot be said for low-income sub-Saharan populations. Reports from different sub-Saharan populations vary, but the overriding consensus appears to be that the prevalence of malnutrition is exacerbated in HIV and AIDS due to factors such as lack of employment income, stigmatisation and other social determinants that contribute to household food insecurity [28–34]. In the present study, maize meal represented the staple diet for the majority of patients—a diet shown in a previous South African study to have a negative impact on the nutritional status of HIV-infected patients [35]. In view of the prevalence of malnutrition in the general population and the contribution of HIV and AIDS to household food insecurity, it seems feasible to assume that malnutrition may be a contributor to the differences found between tryptophan levels in the patients of this study and those from developed countries.

### Higher inflammatory activity as a contributor to a greater degree of tryptophan depletion in sub-Saharan populations

Oxidation in the kynurenine pathway, under influence of the enzyme IDO, is primarily driven by pro-inflammatory activity [7, 13]. When the levels of pro-inflammatory activity, i.e. neopterin, in this study were compared to that of HIV populations from the developed world, it was seen that, at comparable CD4 counts, neopterin levels were much higher in our patients (Table 3). This and the inverse correlations found between neopterin and tryptophan levels (*r* = −0.399; *p* = 0.0001) thus support the notion of higher pro-inflammatory activity, and by implication tryptophan oxidation, at corresponding levels of immune deficiency.

There are two feasible reasons for higher pro-inflammatory activity in low-income sub-Saharan, and more specific, low-income South African populations, than in populations from the developed world. The first would be a higher prevalence of infections, including subclinical infections, and the second a higher prevalence of malnutrition, coupled to the effect of malnutrition on the immune system.

The high prevalence of infections in sub-Saharan countries is well-known [36]. Factors that contribute to higher infection-related inflammatory activity include the presence of untreated clinical and subclinical opportunistic infections, higher levels of health-care-associated (nosocomial) infections, lower availability of medical resources, as well as variable accessibility and adherence to medications [37–42]. While the physician/patient ratio in the developed world varies around 4 doctors per 1000 patients, the South African ratio is estimated to be about 0.8 doctors per 1000 patients, with even lower ratios at state hospitals [43, 44]. Another factor that may have an influence is the stage of the disease at which HAART is initiated [45]. In contrast to American guidelines which suggest initiation of HAART at CD4 < 500 cells/μl, the South African Government guidelines recommend initiation at CD4 cell counts <350 cells/μl [46, 47].

The second potential contributor to a higher level of inflammatory activity, in the low-income population of this study, is the effect of malnutrition on the immune system [48]. It is known that malnutrition can lead to increases in inflammatory mediators such as pro-inflammatory cytokines and in the levels of the acute phase protein CRP [48].

### Tryptophan depletion is part of the general immune-induced effects on the nutritional status of HIV infection and AIDS

The effects of inflammation on nutritional status, i.e., disease-related malnutrition, as well as the low responsiveness of disease-related malnutrition to nutrient supplementation, are well-described in a number of recent publications [49–52]. Although a multitude of studies have been published on the nutritional status of patients with HIV infection and AIDS [23, 53], the fact that most indicators of nutritional status are adversely influenced by the activity of pro-inflammatory cytokines [49, 51, 54–56] is generally overlooked. The malnutrition profile is thus often described only in terms of food insecurity. In the present study, the effects of HIV-induced inflammation on indicators of the nutritional profile, such as albumin, haemoglobin, the albumin/globulin (A/G) ratio, red cell distribution width (RDW) and BMI [49, 51, 54–56], are confirmed by the negative correlations found between the levels of these indicators and that of the pro-inflammatory markers neopterin, IL-6 and CRP (Table 2). Tryptophan levels, as discussed in earlier paragraphs, similarly declined with increases in inflammatory activity. Moreover, highly significant correlations were seen between tryptophan levels and levels of the nutritional markers albumin, haemoglobin, the A/G ratio and RDW (Table 2)—implying associations between the inflammation-induced disturbance in tryptophan levels and that of known markers of nutritional status. We thus contend that tryptophan depletion as a result of increased oxidation in the kynurenine pathway in HIV-infected patients forms part of the general immune-induced alterations in the nutritional status.

## Conclusions

Tryptophan levels in HIV-infected and AIDS patients are markedly lower in low-income, sub-Saharan HIV-infected populations than in populations from developed countries. Food insecurity and higher levels of inflammatory activity are the most probable causes. We contend that tryptophan depletion, due to oxidation in the kynurenine pathway, should be seen as part of the much wider effect of pro-inflammatory activity on the nutritional profile of HIV-infected patients.
